# The epidemiology, healthcare and societal burden and costs of asthma in the UK and its member nations: analyses of standalone and linked national databases

**DOI:** 10.1186/s12916-016-0657-8

**Published:** 2016-08-29

**Authors:** Mome Mukherjee, Andrew Stoddart, Ramyani P. Gupta, Bright I. Nwaru, Angela Farr, Martin Heaven, Deborah Fitzsimmons, Amrita Bandyopadhyay, Chantelle Aftab, Colin R. Simpson, Ronan A. Lyons, Colin Fischbacher, Christopher Dibben, Michael D. Shields, Ceri J. Phillips, David P. Strachan, Gwyneth A. Davies, Brian McKinstry, Aziz Sheikh

**Affiliations:** 1 Asthma UK Centre for Applied Research, Centre for Medical Informatics, Usher Institute of Population Health Sciences and Informatics, The University of Edinburgh, Edinburgh, EH8 9AG UK; 2 Edinburgh Clinical Trials Unit, Centre for Medical Informatics, Usher Institute of Population Health Sciences and Informatics, The University of Edinburgh, Edinburgh, EH8 9AG UK; 3 Population Health Research Institute, St George’s, University of London, Cranmer Terrace, London, SW17 0RE UK; 4 School of Health Sciences, University of Tampere, 33014 Tampere, Finland; 5 Swansea Centre for Health Economics (SCHE), College of Human and Health Science, Swansea University, Singleton Park, Swansea, SA2 8PP UK; 6 Farr Institute, Swansea University Medical School, Singleton Park, Swansea, SA2 8PP UK; 7 The University of Edinburgh & The Royal College of Surgeons of Edinburgh, Clinical Surgery, Royal Infirmary of Edinburgh, Edinburgh, EH16 4SA UK; 8 Information Services Division (ISD), NHS National Services Scotland, Room 111, Gyle Square, 1 South Gyle Crescent, Edinburgh, EH12 9EB UK; 9 School of Geosciences, The University of Edinburgh, Edinburgh, EH8 9AG UK; 10 Centre for Experimental Medicine, School of Medicine, Dentistry and Biomedical Sciences, Wellcome Wolfson Institute Experimental Medicine, Queen’s University Belfast, 97 Lisburn Road, Belfast, BT9 7BL UK; 11 Asthma & Allergy Group, Institute of Life Science, Swansea University Medical School, Swansea University, Singleton Park, Swansea, SA2 8PP UK

**Keywords:** Asthma, Epidemiology, Burden, Cost, UK

## Abstract

**Background:**

There are a lack of reliable data on the epidemiology and associated burden and costs of asthma. We sought to provide the first UK-wide estimates of the epidemiology, healthcare utilisation and costs of asthma.

**Methods:**

We obtained and analysed asthma-relevant data from 27 datasets: these comprised national health surveys for 2010–11, and routine administrative, health and social care datasets for 2011–12; 2011–12 costs were estimated in pounds sterling using economic modelling.

**Results:**

The prevalence of asthma depended on the definition and data source used. The UK lifetime prevalence of patient-reported symptoms suggestive of asthma was 29.5 % (95 % CI, 27.7–31.3; n = 18.5 million (m) people) and 15.6 % (14.3–16.9, n = 9.8 m) for patient-reported clinician-diagnosed asthma. The annual prevalence of patient-reported clinician-diagnosed-and-treated asthma was 9.6 % (8.9–10.3, n = 6.0 m) and of clinician-reported, diagnosed-and-treated asthma 5.7 % (5.7–5.7; n = 3.6 m). Asthma resulted in at least 6.3 m primary care consultations, 93,000 hospital in-patient episodes, 1800 intensive-care unit episodes and 36,800 disability living allowance claims. The costs of asthma were estimated at least £1.1 billion: 74 % of these costs were for provision of primary care services (60 % prescribing, 14 % consultations), 13 % for disability claims, and 12 % for hospital care. There were 1160 asthma deaths.

**Conclusions:**

Asthma is very common and is responsible for considerable morbidity, healthcare utilisation and financial costs to the UK public sector. Greater policy focus on primary care provision is needed to reduce the risk of asthma exacerbations, hospitalisations and deaths, and reduce costs.

**Electronic supplementary material:**

The online version of this article (doi:10.1186/s12916-016-0657-8) contains supplementary material, which is available to authorized users.

## Background

Asthma is now one of the most common long-term conditions in the world [1, 2]. Our previous related work, commissioned to inform United Kingdom (UK) and Scottish parliamentary reviews of allergy services, demonstrated that the UK had amongst the highest prevalence of allergy and asthma in the world [2–4]. This work was important in influencing a range of national policy developments, but is now dated. Given that asthma was highlighted as the major contributor to the estimated burden and costs, there is a particular need for a more up-to-date and detailed review of the burden, healthcare utilisation and costs, and outcomes of asthma [5]. This need was underscored by the recent National Review of Asthma Deaths, which concluded that “*46 % of asthma deaths could have been avoided with better routine care*” [6].

In undertaking the present study, we sought to overcome important limitations of our previous studies [2–4] by extending the scope from healthcare costs alone to include wider societal costs and by incorporating data from previously unavailable datasets; these included out-of-hours care, ambulance, accident and emergency (A&E), intensive care unit (ICU) utilisation, and disability living allowance (DLA) data.

In this paper, we describe the overall epidemiology, healthcare utilisation and costs of asthma for the UK as a whole and, for the first time, its member countries (i.e. England, Scotland, Wales and Northern Ireland).

## Methods

### Overview of methods

Our methods have been described in detail in our published protocol [7].

We undertook secondary analyses of national health surveys, primary and secondary National Health Service (NHS) datasets, and national administrative data. In instances where relevant data were unavailable from a single source, datasets were linked. Overall, we analysed data from 27 datasets, of which five were linked. The data sources used to measure the study outcomes for each country are shown in Table 1. We analysed and reported the findings for 2011–12, or where this was not available, for 2010–11. The base year used for all costs was the 2011–12 financial year, applying appropriate inflation indices where required [8].

**Table 1:**
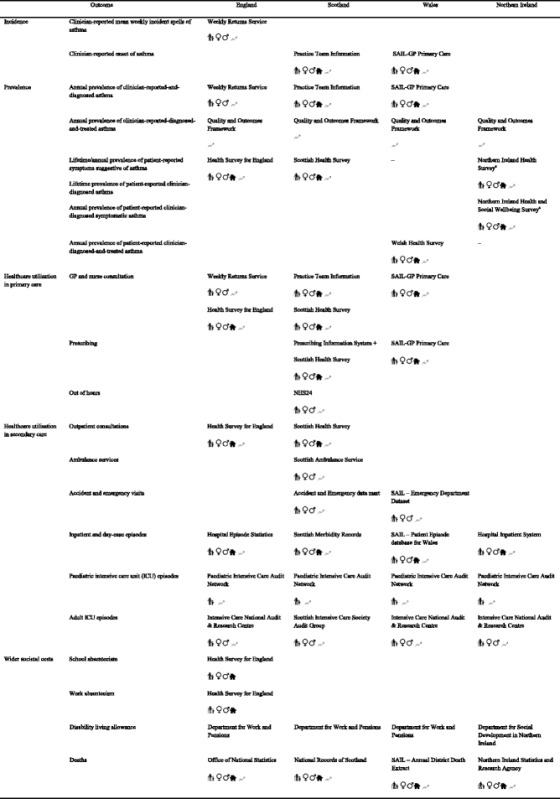
Study outcomes with datasets used, demographic and time-trend information availability therein, by UK nation

Blank cells indicate unavailability of data source for the needs and scope of our study

^a^Due to nature of data collection, data could not be standardised for all ages

Symbols represent:  age, sex,  socioeconomic status,  time trend

SAIL, Secure Anonymised Information Linkage

### Study population

The denominator for each dataset was based on the total sample of people in the dataset or the mid-year population estimate of the country where the dataset covered the entire population. The mid-year UK population estimates were 62,759,456 in 2010–11 and 63,285,100 in 2011–12 [9, 10].

### Study outcomes

Depending on the dataset, patients with asthma were defined as follows:

1. Being diagnosed with asthma in primary care based on relevant Read codes (Additional file 1: Appendix 1) [7]
2. Respondents in national health surveys who reported symptoms or treatment suggestive of asthma or reported clinician-diagnosed asthma [7]
3. Having received asthma medications prescribed by their general practitioner (GP) for asthma, where prescriptions were coded using British National Formulary (BNF) codes (Additional file 1: Appendix 2) [7, 11]
4. Using NHS out-patient clinic, out-of-hours service, ambulance service or A&E for asthma
5. Having a primary diagnosis of asthma with ICD-10 code of J45 for asthma or J46 for status asthmaticus at discharge from hospital [7, 12]
6. Having a primary diagnosis of asthma at admission with Read codes (Additional file 1: Appendix 3) in paediatric ICU and ICD-10 codes J45 or J46 or Acute Physiology and Chronic Health Evaluation (APACHE) III diagnostic code for asthma in adult ICUs [7, 13]
7. Having ICD-10 codes J45 or J46 for asthma as the main disabling condition for claiming DLA or as the underlying cause of death at registration [12, 14]

Thus patients or events where the main reason for healthcare or societal care utilisation was asthma were all included. This criterion thus (1) includes patients with asthma who might have had comorbidities, but only their asthma was accounted for and not their comorbidities, (2) does not include patients where asthma was not the main diagnosis.

### Outcome measures and datasets used

The data sources used in the respective countries to assess the outcome measures along with availability of demographic information used and time trend by UK nations is presented in Table 1.

### Incidence

Our primary aim was to measure healthcare utilisation; therefore, our focus was to estimate asthma incident-spells that generated a contact with primary care (see description for England below). Secondarily, where possible, we also estimated the incidence of first occurrence of asthma (incident cases) (see description for Scotland and Wales below). However, considering that an asthma episode may present in secondary care and that most UK primary and secondary care data are not linked, it was challenging to identify with certainty if an asthma episode presenting in secondary care represented the first occurrence of the asthma case. Due to differences among data sources and reporting, we used two measures of incidence, namely (1) clinician-reported mean weekly incident spells of asthma and (2) clinician-reported onset of asthma. In England, weekly incidence of asthma episodes was estimated from averaging new weekly episodes recorded by the Weekly Returns Service (WRS) of the Royal College of General Practitioners [15]. WRS receives notifications of weekly episodes and numbers of consultations for asthma using ICD-9 code 493, from about 90 general practices covering over 800,000 people in England. WRS episodes are available by age-groups and sex for each quarter and year.

In Scotland, Practice Team Information (PTI) was used to measure onset of asthma resulting in new GP consultation [16]. PTI is a GP-database comprising a sample of 60 general practices representing about 6 % of Scottish general practices and around 6 % of the Scottish patient population. It includes GP and nurse consultations and diagnoses using Read codes, along with demographic data. PTI was established in 2003–04 and we used this year as the starting point of follow-up for 5 years. Onset of asthma was defined as new GP consultation in patients who were consistently in PTI since 2003–04 and did not consult their GP for asthma for those 5 years, but consulted their GP for asthma after 2008–09. This assumes that patients who consulted their GP for asthma before 2003 would come to see their GP at least once in those 5 years. Following this method, only new consultations which had a Read code for asthma in 2011–12 were counted.

In Wales, onset of asthma resulting in new GP consultation was estimated from the Secure Anonymised Information Linkage (SAIL) databank, which during the study period collected data from 42 % of the GP practices in Wales [17]. There were data available on demographics and diagnoses based on Read codes (Additional file 1: Appendix 1). Only patients who had not deregistered from the participating GP practices and did not consult a GP for asthma between 2006–07 and 2010–11 and had new consultations with Read code for asthma (Additional file 1: Appendix 1) in 2011–12 were counted.

We could not identify any GP-database in Northern Ireland that could be used to estimate annual onset of asthma by new GP consultation within the available budget for this work.

### Prevalence

We defined annual prevalence as the proportion of the population who experienced symptoms of asthma at least once during the study year and life prevalence as the proportion of the population who experienced symptoms of asthma for at least part of their lives at any time during their life course [18]. Besides using lifetime and annual prevalence, we distinguished between patient-reported and clinician-reported measures, when the data pertained to health surveys and primary care data recorded by GPs, respectively. Thus, we used seven measures of prevalence, of which (1) lifetime prevalence of patient-reported symptoms suggestive of asthma, (2) annual prevalence of patient-reported symptoms suggestive of asthma, (3) lifetime prevalence of patient-reported clinician-diagnosed asthma, (4) annual prevalence of patient-reported clinician-diagnosed symptomatic asthma, and (5) annual prevalence of patient-reported clinician-diagnosed-and-treated asthma were based on national health surveys and (6) annual prevalence of clinician-reported-and-diagnosed asthma and (7) annual prevalence of clinician-reported-diagnosed-and-treated asthma were based on primary care data. The health surveys used were the Health Survey for England (HSE) [19], Scottish Health Survey (SHeS) [20], Welsh Health Survey (WHS) [21] and Northern Ireland Health Survey [22]. These surveys were of randomly selected samples of people broadly representative of their respective general populations. They included information on self-reported health and utilisation of health services. While the questions for asthma in the national health surveys were similar in England and Scotland, in Wales, only one question was asked questioning whether the respondent had been treated for asthma in that year [23]. Thus, only annual prevalence of patient-reported clinician-diagnosed-and-treated asthma could be estimated for Wales using the national health survey. Northern Ireland Health Survey asthma data were mainly on adult respondents, since information on children of ages between 2 and 14 years were grouped together. We thus could not use this information on children on age standardisation and hence national estimates could not be ascertained for Northern Ireland using national health survey data [23].

The prevalence estimates from primary care databases came from WRS in England [15], PTI in Scotland [16] and SAIL-GP in Wales [17], all of which had individual-level data. Additional estimates came from the Quality and Outcomes Framework (QOF) data, which was the only data source available from all four UK nations [24–27]; this was, however, aggregated at GP practice level, so could not provide a breakdown by age and sex [7]. QOF data pertaining to asthma (which is one of the many indicators) are a count of all people of all ages with asthma registered with GP practices, excluding patients with asthma who were not prescribed asthma-related drugs in the last 12 months (Quality Improvement code Asthma 1) [28]. We were unable to identify any suitable primary care data source from Northern Ireland within our budget.

The definitions for the prevalence measures used were:

1. Lifetime prevalence of patient-reported symptoms suggestive of asthma – defined as the number of people who had responded yes to “Have you had wheezing or whistling in the chest at any time, either now/in the past?” in HSE for England or in SHeS for Scotland, divided by the number of respondents who had answered that question in HSE or SHeS for England and Scotland, respectively.
2. Annual prevalence of patient-reported symptoms suggestive of asthma – defined as the number of people who had responded yes to “Have you had wheezing or whistling in the chest in the last 12 months?” in HSE for England or in SHeS for Scotland, divided by the number of respondents who had answered that question in HSE or SHeS, for England and Scotland, respectively.
3. Lifetime prevalence of patient-reported clinician-diagnosed asthma – for England, defined as the number of people who had responded yes to “Did a doctor or nurse ever tell you that you had asthma?” in HSE divided by the number of respondents who had answered that question, and for Scotland “Did a doctor ever tell you that you had asthma?” in SHeS, divided by the number of respondents who had answered that question.
4. Annual prevalence of patient-reported clinician-diagnosed symptomatic asthma – for England, defined as the number of people who had responded yes to both the questions “Have you had wheezing or whistling in the chest in the last 12 months?” and “Did a doctor or nurse ever tell you that you had asthma?” in HSE, divided by the number of respondents who had answered the former question. For Scotland, the number of people who had responded yes to both the questions “Have you had wheezing or whistling in the chest in the last 12 months?” and “Did a doctor ever tell you that you had asthma?” in SHeS, divided by the number of respondents who had answered the former question.
5. Annual prevalence of patient-reported clinician-diagnosed-and-treated asthma – defined as the number of people who had responded yes to both the questions “Did a doctor or nurse ever tell you that you had asthma?” and “Over the last 12 months, have you used an inhaler/puffer/nebuliser prescribed by a doctor to treat your asthma/wheezing/whistling/difficulty in breathing?” in HSE, divided by the number of respondents who had answered the latter question for England. For Scotland, the number of people who had responded yes to both the questions “Did a doctor ever tell you that you had asthma?” and “Were you treated in the past 12 months for wheeze by GP/nurse at surgery/community/school/district nurse/hospital, consultant/specialist at hospital, consultant/specialist elsewhere, homeopath/acupuncturist/other alternative medicine professional” in SHeS, divided by the number of respondents who had answered the latter question. For Wales, the only available question used was “are you currently being treated for asthma” in WHS, with the numerator as the number of respondents who said yes to that and the denominator as the total number of respondents to that question.
6. Annual prevalence of clinician-reported-and-diagnosed asthma – based on PTI’s Read code grouping ‘asthma’ (Additional file 1: Appendix 1) and obtained from 39 practices participating in PTI in 2011–12 who had submitted complete GP and practice-nurse data in Scotland and from the GP practices that participated in SAIL-GP in Wales in 2011–12. PTI data were broadly representative of the Scottish population, and so is the population covered by the GP practices representing the Welsh population, thus these estimates are generalizable for Scotland and Wales.
7. Annual prevalence of clinician-reported-diagnosed-and-treated asthma – defined as the proportion of people of all ages who were prescribed asthma-related drugs by GPs for their symptoms of asthma in the last 12 months (Quality Improvement code Asthma 1) [28], compared to the population size the GP practice covered in that year (the list size). Since age and sex are not available in QOF, age standardised rate could not be reported and thus only crude rates are presented.

### Healthcare utilisation in primary care

#### GP and nurse consultation

For estimating GP and nurse consultations for asthma, WRS was used for England, PTI for Scotland and SAIL-GP for Wales. WRS had ICD-9 codes, PTI and SAIL-GP had Read codes (Additional file 1: Appendix 1).

#### Prescriptions

Community prescriptions for asthma were identified from the SAIL-GP database based on our list of BNF codes (Additional file 1: Appendix 2) [7]. Some medications indicated for use in asthma can also be prescribed to treat other conditions, primarily chronic obstructive pulmonary disease (COPD). We therefore confined our analysis of medications to people with a clinician-recorded diagnosis of asthma, but without a diagnosis of COPD [7]. To address this issue, medications of patients who had COPD and asthma were included so long as they received the list of asthma medication we had (Additional file 1: Appendix 2), but patients with COPD but no asthma were excluded.

For comparison, the total cost of all medications used for asthma (excluding immunosuppressants, which are also used for other conditions) was also examined in Prescription Costs Analysis (PCA) data for each of the four countries [29–32]. It should be noted that, because PCA data did not include information on diagnosis, these data included medications with an indication for asthma, but which were prescribed for other conditions. In pre-specified sensitivity analyses, community prescribing costs for Scottish patients under the age of 40 (in whom COPD is rare), extracted from Scottish Prescribing Information Systems (PIS) data, were compared to the figures extrapolated from the SAIL-GP results to test the reliability of the extrapolation process.

#### Out-of-hours

Information on calls to an out-of-hours NHS service were only obtainable from NHS 24 Scotland, the national telephone triage and advice service [33]. Data are available from 2008 onwards. All calls where the nurse triaging the out-of-hours call selected an asthma-specific algorithm to support their decision-making were collected. In England, although an out-of-hours surveillance team exists, a breakdown by asthma was not available. The out-of-hours data in Wales were inconsistently collected across areas and hence were not used. We could get out-of-hours data on asthma for Northern Ireland.

### Healthcare utilisation in secondary care

#### Out-patient attendances

Although routine data on attendances in NHS out-patient clinics were available across the four nations, these data were, however, captured under the broader heading of ‘respiratory’ consultations and it was therefore not possible to estimate the proportions of these consultations that were particularly for asthma. This is noted as a major data gap.

#### Ambulance services

For Scotland, asthma data from the Scottish Ambulance Service, which had data from 2008–09 onwards, were used where the record had “Emergency call-asthma selected” [34]. Usage of ambulance service due to asthma could not reliably be estimated from the aggregated routinely collected data available in England, Wales and Northern Ireland; this is therefore identified as a data gap.

#### A&E services

In England, there were no accurate published data on A&E attendances for asthma. In Scotland, the A&E data mart was used for sites which reported (excluding Orkney and Tayside Health Boards, since they currently only submit high level diagnosis codes to ISD) patient-level information from their A&E departments since 2010–11 [35]. The data reported here are from ‘new’ and ‘unplanned return’ attendances at A&E, i.e. excluding those who were ‘recall’ or ‘planned return’. If the ‘disease code’ included the ICD-10 codes above or ‘R062’ (Family history of asthma) or if the ‘presenting complaint text’ or ‘diagnosis text’ referred to any of the terms asthma, wheezing, low saturation, chest tightness or shortness of breath, then those cases were selected. In Wales, the SAIL Emergency Department Dataset, which contains data since 2009, were used [36]. There was an audit data in Northern Ireland on A&E in one of its Trusts, namely Belfast Health and Social Care Trust, which collected data from 2007–08 onwards. Since that data from one urban Trust may not have been representative of the entire nation, we did not use these for our national estimates.

#### Inpatient and day cases in hospitals

We queried the Hospital Episode Statistics for England [37], General/Acute Inpatient and Day Case dataset for Scotland [38], SAIL Patient Episode Database for Wales [39], and Hospital Inpatient System dataset in Northern Ireland [40], for primary diagnosis of asthma with ICD-10 codes to identify all asthma episodes. However, hospital-based prescribing was not included in these datasets.

#### ICUs

The Paediatric Intensive Care Audit Network (PICA Net) is a national audit which collects data on all critically ill children admitted to paediatric ICUs across the UK [41]. It had data from England and Wales from 2002, from Scotland from 2007, and from Northern Ireland from 2008 onwards, recorded in Read version 3 (Additional file 1: Appendix 3). For adults in ICUs, for England, Wales and Northern Ireland, we used Intensive Care National Audit & Research Centre (ICNARC) data, which have been collected since 1996 and which uses ICD-10 codes [42]. For Scotland, the Scottish Intensive Care Society Audit Group (SICSAG) data were queried and APACHE III diagnoses for asthma was used [13, 43]. For all of these countries, data from stand-alone ICUs or from ICUs mixed with high dependency units were included. Data from stand-alone high dependency units were excluded.

### Wider societal impact

To capture impact beyond the health services, we investigated absenteeism in school and at work, care-at-home, DLA and mortality.

#### Absenteeism

School and work absenteeism data were obtained from HSE 2010 for England, from the questions “Over the last 12 months, how many days has your asthma/wheezing/whistling in (your/his/her) chest caused (you/him/her) to be absent from school?” and “Over the last 12 months, how many days has your wheezing/whistling in your chest, shortness of breath or difficulty in breathing caused you to be absent from work?”, respectively, among asthma respondents. We could not identify any suitable data source to investigate school and work absenteeism in Scotland, Wales or Northern Ireland. Since HSE reports absence as a categorical variable: < 5 days, 5–9, etc., we used mid-points to estimate the number of days. The estimates produced were for the number of days of absence as a proportion of the sample population, so the rates were of number of days of absence per 1000 population.

#### Care-at-home

We were unable to identify any suitable data to estimate costs of care at home for asthma from any nation.

#### DLA

Aggregated data were available from the Department of Work and Pensions (DWP) [14], the government agency providing national benefits on number of people receiving DLA and DLA amount, with asthma as the main disabling condition in England, Scotland and Wales for 2011–12. For Northern Ireland, there were data available from the Department for Communities on number of people receiving DLA due to asthma as the main disabling condition and total amount by age-group, sex and SES from 2008 [44].

#### Premature retirement

We could not identify a data source for this outcome and therefore identified this as a data gap.

#### Mortality

Mortality data with underlying cause of death as asthma from death certificate registrations, coded using ICD-10, are available from the Office of National Statistics for England and Wales [45], National Records Scotland [46], and Northern Ireland Statistics and Research Agency for Northern Ireland [47] were queried.

### Analyses

Counts of events or people, as the case was, were obtained across all the age-groups (<5, 5–9, 10–14, … 70–74, > 75 years) (except QOF) for that year, along with the denominator. For comparison across nations, figures obtained across the datasets were age standardised using the European Standard Population (Version 2013) [48]. Age-standardised epidemiological, healthcare utilisation, school and work absenteeism and DLA estimates, accompanied by their respective 95 % confidence intervals (CIs) were reported based on the Poisson approximation [49]. UK-wide summaries of incidence and prevalence estimates and associated 95 % CIs were calculated by inverse variance, fixed effect meta-analyses in R (Version 3.1.0).

Healthcare costs were estimated from a NHS perspective based on healthcare utilisation using NHS data, detailed in Additional file 1: Appendix 4. Where a given dataset did not include a direct measure of costs, standard UK price weights were applied to generate cost estimates for each form of healthcare [8, 11, 50]. Additional file 1: Appendix 4 summarises the costing method applied in each case. Primary care price weights were taken from the Personal and Social Services Research Unit. Community prescribing costs were based on net ingredient costs based on SAIL-GP prescribing for asthma medications in non-COPD patients [11, 17]. These data contained details of the type and date of medication prescribed, but not the number of items prescribed on a given date. Therefore, a conservative assumption that a single pack was prescribed at each time had to be applied, which underestimated the costs associated with prescriptions for multiple items. Inpatient care costs were based on NHS Reference Cost estimates based on their associated Healthcare Resource Grouping (Version 4) [50].

Societal costs were estimated from a wider societal perspective, including NHS costs as above and DLA. Though we originally aimed to include productivity costs, it was not possible to reliably estimate costs due to school and work absenteeism since the data were not asthma specific and excluded some key variables.

### Addressing data gaps for cost analysis

Data gaps found were of three forms: (1) within country, where no single dataset in a given country held sufficient variables to provide an estimate, but linkages between datasets could overcome this; (2) between countries, where data on the variable of interest (after allowing for linkages) were available in one member country, but not in another; and (3) across countries, where no data (after allowing for linkages) from any member country were found for the variables of interest.

For type (1) data gaps, linked data were used. For type (2) data gaps, estimates of a given variable from one country, where available (e.g. prescription costs in Wales), were mapped onto other countries, adjusting for population size, annual prevalence of clinician-reported-diagnosed-and-treated asthma (QOF), which was available across all the nations, and the age and sex distribution of patients who reported having asthma in the respective country’s health survey, or in the SAIL-GP database (Additional file 1: Appendix 5). For type (3) data gaps, a literature search was undertaken in an attempt to provide parameter estimates for modelling (although no usable data-fitting modelling requirements were found).

### Economic modelling

An economic model of the costs of asthma in the UK and its member countries was built in Excel 2010 (Additional file 1: Appendix 6). In brief, data on resource use were taken directly from healthcare utilisation data based on the internal diagnostic coding (such as Read code or ICD-10) available in each dataset. For factors such as hospital episodes and DLA, whole population datasets were available and complete, thus not requiring any adjustment. The model applied the price weights detailed above to generate costs from this resource use [8, 11, 50]. Costs based on a sample within a country were extrapolated to population levels by rescaling per head of age-sex stratified population. Where results were extrapolated to another country due to data gaps, additional rescaling was undertaken based on each country’s relative QOF prevalence, in order to account for differences in prevalence rates between countries [25–28]. QOF was selected for this process due to its relationship to treated asthma, which provided the most appropriate data source for healthcare utilisation and was the only measure of prevalence measured in a uniform manner in all countries. The model was also used to sum the cost estimates into any required groupings; bootstrapping (with 10,000 replicates) was used to estimate 95 % CIs around the joint distributions of each total cost estimate using the percentile method (Additional file 1: Appendix 6) [51]. Following recommendations in standard modelling guidance [52], the uncertainty around prevalence estimates were simulated using a beta distribution and uncertainty around cost estimates were simulated using a gamma distribution or a normal distribution where sample sizes were large and central limit theorem was expected to hold (Additional file 1: Appendix 6).

## Results

The data below refer to UK-wide estimates unless otherwise stated.

### Incidence

We estimated that the annual age-standardised incidence of GP-diagnosed asthma was 3.8/1000 (95 % CI, 3.8–3.9), equivalent to approximately 240,000 people in the UK developing asthma in 2011–12. On average, there were 5600 weekly incident GP episodes of asthma (Table 2).

**Table 2:** Incidence and prevalence of asthma in patients of all ages by UK nation

Epidemiologic measures		England		Scotland		Wales		Northern Ireland		UK estimate (inverse variance, fixed effect meta-analysis)	
		n	ASR^i^	n	ASR^i^	n	ASR^i^	n	ASR	n	ASR
		N	(95 % CI)	N	(95 % CI)	N	(95 % CI)	N	(95 % CI)	N	(95 % CI)
Incidence/1000 in 2011–12	Clinician-reported onset of asthma^1,2,a,b,c^			20,780	3.8	4779	3.7			240,483	3.8
				5,511,732	(3.7–3.9)	1,108,024	(3.6–3.9)			63,285,100	3.8–3.9
	Clinician-reported mean weekly incident-spells of asthma^3,a^	77	0.1							5,696	0.1
		722,885	(0.1–0.1)							63,285,100	0.1–0.1
Prevalence/100 patient-reported in 2010–11 and clinician-reported in 2011–12	Lifetime prevalence of patient-reported symptoms suggestive of asthma^4,5,d^	4335	31.3	794	24.6					18,514,040	29.5
		14,112	(30.2–32.4)	3256	(22.9–26.4)					62,759,456	27.7–31.3
	Annual prevalence of patient-reported symptoms suggestive of asthma^4,5,e^	2465	18.0	489	14.8					10,731,867	17.1
		14,112	(17.1–18.8)	3256	(13.4–16.2)					62,759,456	15.7–18.5
	Lifetime prevalence of patient-reported clinician-diagnosed asthma^4,5,f^	2280	16.1	443	14.0					9,790,475	15.6
		14,112	(15.3–16.9)	3256	(12.6–15.3)					62,759,456	14.3–16.9
	Annual prevalence of patient-reported clinician-diagnosed symptomatic asthma^4,5,g^	1235	8.6	229	7.0					5,083,516	8.1
		14,112	(8.0–9.1)	3256	(6.1–7.9)					62,759,456	7.2–9.1
	Annual prevalence of patient-reported clinician-diagnosed-and-treated asthma^4,5,6,h^	1320	9.3	322	9.8	1901	9.8			6,024,908	9.6
		14,112	(8.6–9.9)	3255	(8.7–11.0)	19,225	(9.4–10.4)			62,759,456	8.9-10.3
	Annual prevalence of clinician-reported-and-diagnosed asthma^1,2,a,b,c^			310,050	5.7	63,873	5.7			3,600,861	5.7
				5,511,732	(5.7–5.7)	1,119,368	(5.7–5.8)			63,285,145	5.7–5.7
	Annual prevalence of clinician-reported-diagnosed-and-treated asthma^7,ii^ *Crude rate*	3,295,944	*5.9*	319,091	*6.0*	218,243	*6.9*	113,518	*6.0*	4,303,390	6.8
		55,525,732	*(5.9–5.9)*	5,299,097	*(6.0–6.0)*	3,185,538	*(6.9–6.9)*	1,898,678	*(5.9–6.0)*	63,285,145	6.8–6.8

*Source*: ^1^Practice Team Information (PTI), Scotland; ^2^Secure Anonymised Information Linkage-GP, Wales; Health Survey for England 2010; ^**3**^Weekly Returns Service, England; ^4^Health Survey for England, ^5^Scottish Health Survey 2010; ^6^Welsh Health Survey, 2010; ^7^Quality Outcomes Framework (QOF)

^i^Age standardised rate (ASR)

^ii^Since age and sex are not available in QOF, crude rate is presented

Blank cells had no data availability

^a^Based on ISD’s Read Code Grouping ‘Asthma’

^b^PTI estimates are based on 40, 43, 39 and 39 practices that submitted complete GP and practice-nurse data over a 6-year period ending 31 March 2009, 2010, 2011 and 2012, respectively. PTI data are broadly representative of the Scottish population

^c^The Welsh estimates apply to GP practice areas that participate in SAIL-GP. Population covered by these GP practices represent the Welsh population, thus these estimates are generalizable for Wales

^d,e,f,g,h^Prevalence estimates were derived from questions in repeated population health surveys of the respective UK nations

^d^“Have you ever had wheezing/whistling in the chest at any time, either now/in the past?” in England and Scotland

^e^“Have you had wheezing or whistling in the chest in the last 12 months?” in England and Scotland

^f^England – “Did a doctor or nurse ever tell you that you had asthma?”; Scotland – “Did a doctor ever tell you that you had asthma?”; Wales – there was no equivalent question asked in the survey from Wales

^g^Questions in ^e^ and ^f^

^h^Questions in ^f^ AND “Over the last 12 months, have you used an inhaler/puffer/nebuliser prescribed by a doctor to treat your asthma/wheezing/whistling/difficulty in breathing?” for England, “Were you treated in the past 12 months for wheeze by GP/nurse at surgery/community/school/district nurse/hospital, consultant/specialist at hospital, consultant/specialist elsewhere, homeopath/acupuncturist/other alternative medicine professional?” for Scotland, and “Are you currently being treated for asthma?” for Wales

### Prevalence

The lifetime prevalence of patient-reported symptoms suggestive of asthma was 29.5 % (95 % CI, 27.7–31.3), equivalent to 18.5 m people. The annual prevalence of patient-reported symptoms suggestive of asthma was 17.1 % (95 % CI, 15.7–18.5), equivalent to 10.7 m people (Table 2).

The lifetime prevalence of patient-reported clinician-diagnosed asthma was 15.6 % (95 % CI, 14.3–16.9), equivalent to 9.8 m people; annual prevalence of patient-reported clinician-diagnosed symptomatic asthma was 8.1 % (95 % CI, 7.2–9.1), which equated to 5.1 m people; annual prevalence of patient-reported clinician-diagnosed-and-treated asthma was 9.6 % (95 % CI, 8.9-10.3), which equated to 6.0 m people; annual prevalence of clinician-reported-and-diagnosed asthma was 5.7 % (95 % CI, 5.7–5.7), which equated to 3.6 m people; clinician-reported-diagnosed-and-treated asthma was 6.8 % (95 % CI, 6.8–6.8), which equated to 4.3 m people.

### Healthcare utilisation in primary care

There were an estimated 2.7 m (95 % CI, 2.6–3.0) GP consultations, 3.7 m (95 % CI, 3.6–4.1) nurse consultations and 54,000 (95 % CI, 53,000–60,000) out-of-hours calls for asthma (Table 3).

**Table 3:** Healthcare utilisation in primary care for asthma across all ages in 2011–12 by UK nation

Healthcare utilisation measure in primary care	England		Scotland		Wales		Northern Ireland		UK estimate^3^
	n	ASR^a^	n	ASR^a^	n	ASR^a^	n	ASR^a^	n (000 s)
	N	(95 % CI)	N	(95 % CI)	N	(95 % CI)	N	(95 % CI)	(95 % CI)
Number of General Practitioner consultations^1^			215,610	39.1					2700
			5,511,732	(39.0–39.3)					(2600–3029)
Number of nurse consultations^1^			289,120	53.4					3693
			5,511,732	(53.2–53.6)					(3577–4152)
Out of hours calls^2^			4575	0.9					54.3
			5,299,900	(0.8 to 0.9)					(53–60)

*Source*: ^1^Practice Team Information for Scotland; ^2^NHS 24 for Scotland; ^3^from cost modelling

^a^Age standardised rate (ASR) per 1,000 people registered with GP practices in Wales and population for Scotland. Estimates were standardised using the 2013 European Standard Population

Blank cells had no data availability

### Healthcare utilisation in secondary care

There were an estimated 113,000 (95 % CI, 108,000–132,000) ambulance conveyances for asthma; 121,000 (95 % CI, 108,000–146,000) A&E attendances; 93,900 (95 % CI, 93,900–93,900) in-patient episodes; 6100 (95 % CI, 5900–6200) day-case episodes; and 1800 (95 % CI, 1700–1900) ICU episodes (Table 4). The total length of stay for inpatients and day-cases in UK relating to asthma was 195,000 days.

**Table 4:** Healthcare utilisation in secondary care for asthma across all ages in 2011–12 by UK nation

Healthcare utilisation measure in secondary care	England		Scotland		Wales		Northern Ireland		UK estimate^7^
	n	ASR^a^	n	ASR^a^	n	ASR^a^	n	ASR^a^	n (000 s)
	N	(95 % CI)	N	(95 % CI)	N	(95 % CI)	N	(95 % CI)	(95 % CI)
Ambulance conveyance^1^			8263	1.6					112.9
			5,299,900	(1.6–1.7)					(107.6–131.8)
Accident and emergency (A & E) attendances in hospital^2,b^			8457	1.7	2321	0.7			121.1
			4,868,230	(1.6–1.7)	3,033,591	(0.7–0.8)			(108–146)
Inpatient episodes of hospital care (for asthma as the primary reason for care)^3,c^	76,319	1.4	7744	1.5	7887	2.6	1966	1.1	93,916
	53,107,200	(1.4–1.4)	5,299,900	(1.4–1.5)	3,033,591	(2.5–2.7)	1,814,318	(1.0–1.1)	(93,916–93,916)
Day-case episodes of hospital care (for asthma as the primary reason)^3,c^	5066	9.4	142	2.7	768	25.7	144	7.0	6120
	53,107,200	(9.1–9.7)	5,299,900	(2.2–3.1)	3,033,591	23.9–27.6	1,814,318	5.88–8.20	(5929–6248)
Intensive care unit episodes for asthma as the primary reason for care^4,5,6,d^	1537	2.8	179	3.3	97	3.0	55	3.0	1868
	53,107,200	(2.7–3.0)	5,299,900	(2.8–3.8)	11,931,062	(2.4–3.6)	1,704,245	(2.2–3.8)	(1739–1932)

*Source*: ^1^Scottish Ambulance Service (SAS); ^2^A&E data mart in Scotland (excluding Orkney and Tayside Health Boards) and SAIL-Emergency Department Dataset for Wales; ^3^Hospital Episode Statistics-England, General/Acute Inpatient and Day-Case-Scotland, SAIL-Patient Episode Database-Wales and Department of Health, Social Service and Public Safety in Northern Ireland; ^4^For children, Paediatric Intensive Care Audit Network (PICANet); ^5^For adults, Intensive Care National Audit & Research Centre (ICNARC)-England, Northern Ireland and Wales and ^6^Scottish Intensive Care Society Audit (SICSAG)-Scotland; ^7^From cost modelling

^a^Age standardised rate (ASR), using the 2013 European Standard Population; per 1000 population of the country for ambulance, accident and emergency (A&E) and inpatients, and per 100,000 population for day-cases and intensive care

^b^Includes ‘New’ and ‘Unplanned Return’ attendances only at A&E, excludes those who are ‘Recall’ or ‘Planned Return’. For Scotland based on A&E sites which returned episode-level information for at least one of the following: ICD10 Diagnosis code (R098/R068/R062/R060/R05X/R05/J46X/J46/J459/J458/J451/J450/J45/R688/R69X/R69/Z825/J21/J210/J211/J218/J219/R06/R09/R092) OR Diagnosis free-text extracted from “Wheez”/“Asthma”/“Ashtma”/“low” AND “sats”(“chest” AND “tight”) AND (“SOB” OR (“short” AND “breath”)). However, most Health Boards use a pick list/disease code from ICD-10 codes, these are usually mapped from diagnosis text where a pick list has been used. NHS Tayside and NHS Orkney only submit high-level diagnosis codes (comprises about 6 % of total attendance), thus have been excluded here. Thus, figures presented here will be an underestimate of the true number of attendances to A&E for Scotland

^c^ICD-10 codes J45/J46 as primary reason for care. For Wales, the first non-R or Z code in day-cases were also used additionally. R codes refer to “symptoms” and Z codes to “factors influencing health status and contact with health services”

^d^Asthma as primary reason for care with Read codes in PICANet, ICD-10 codes J45/J46 in ICNARC and APACHE III diagnostic codes in SICSAG

Blank cells had no data availability

### Wider societal impact

School absenteeism for asthma or asthma symptoms accounted for 252.4 days/1000 children (95 % CI, 241.3–263.5; n/N = 1267/5352), equivalent to 2.8 m (95 % CI, 2.6–3.0) absences. Work absenteeism for asthma symptoms accounted for 78.9 days/1000 adults (95 % CI, 72.6–85.3, n/N = 535/6978), equivalent to 4.1 m (95 % CI, 3.4–4.7) work-days lost.

For asthma, DLA was claimed by an estimated 36,980 people; 24,100 people in England, 3600 people in Scotland, 3300 people in Wales and 5980 people in Northern Ireland.

There were an estimated 1160 deaths (2.1/100,000; 95 % CI, 2.0–2.2) due to asthma; 982 deaths in England (2.1/100,000; 95 % CI, 2.0–2.2), 94 in Scotland (2.0/100,000; 95 % CI, 1.6–2.3), 58 in Wales (2.0/100,000; 95 % CI, 1.5–2.5), and 26 in Northern Ireland (1.9/100,000; 95 % CI, 1.2–2.7).

### Financial costs of asthma

We estimated that asthma cost at least £1.1 bn with the majority of costs (74 %) arising in primary care, of which 81 % were for community prescribing. Table 5 provides a detailed breakdown of this estimate by member countries and cost elements. For comparison, the total cost of all medications listed in PCA data with an indication for use in asthma (irrespective of condition actually prescribed for) was also £1.1 bn in 2011 (2011–12 for Scotland), of which £821.2 m, £97.5 m, £66.1 m and £38.7 m were incurred in England, Scotland, Wales and Northern Ireland, respectively. It is important to note that the parity between the £1.1 bn total cost estimate from our model and the £1.1 bn total cost of medications with an indication for asthma from PCA data is entirely co-incidental and occurs due to PCA providing an overestimate of medication costs in this context, rather than being a component of the costs used in the model.

**Table 5:** Breakdown of estimated costs for asthma in the UK by member country in 2011–12

	England		Scotland		Wales		Northern Ireland		UK	
	Cost	(95 % CI)	Cost	(95 % CI)	Cost	(95 % CI)	Cost	(95 % CI)	Cost	(95 % CI)
	(£000 s)	(£000 s)	(£000 s)	(£000 s)	(£000 s)	(£000 s)	(£000 s)	(£000 s)	(£000 s)	(£000 s)
GP consultations	89,926	(86,614–101,526)	8624	(8138–9120)	6408	(6116–7411)	3029	(2906–3436)	107,987	(103,986–121,168)
Practice nurse consultations	43,021	(41,614–48,745)	4048	(3876–4213)	3202	(3073–3706)	1431	(1379–1627)	51,702	(50,083–58,131)
Community prescribing	552,514	(536,694–568,687)	54,514	(51,890–57,191)	40,572	(40,178–40,977)	18,845	(18,150–19,504)	666,445	(650,112–683,375)
Calls to out-of-hours	1325	(1291–1485)	130	(130–130)	86	(84–98)	–	–	1541	(1507–1710)
Ambulance Trips	27,511	(26,077–32,480)	2408	(2408–2408)	2378	(2238–2876)	876	(828–1033)	33,172	(31,624–38,649)
Accident and emergency	10,907	(9553–13,357)	913	(913–913)	889	(759–1131)	392	(298–495)	13,101	(11,625–15,782)
Hospital episodes (excluding intensive care units (ICU))	69,162	(69,162–69,162)	6342	(6342–6342)	8128	(8087–8169)	2,064	(2064–2064)	85,696	(85,656–85,737)
ICU episodes	4413	(4413–4413)	482	(482–482)	236	(236–236)	129	(129–129)	5260	(5260–5260)
Total NHS cost	798,780	(780,199–824,168)	77,462	(74,296–79,704)	61,899	(61,141–63,650)	26,764	(25,975–27,772)	964,905	(945,648–991,409)
Disability living allowance	95,500	(95,500–95,500)	14,800	(14,800–14,800)	12,800	(12,800–12,800)	23,832	(23,832–23,832)	146,932	(146,932–146,932)
Total public sector costs	894,280	(880,112–924,082)	92,262	(89,579–94,986)	74,699	(74,177–76,686)	50,596	(49,935–51,732)	1,111,837	(1,097,840–1,143,601)

Please see individual sections of this paper for full commentary and caveats. An important note on the derivation and interpretation of the confidence intervals detailed here is also available in our published protocol

A sensitivity analysis comparing community prescribing costs for Scottish patients under the age of 40 years from PIS data to the costs estimated for the same group extrapolated from SAIL-GP data produced similar figures of £19.2 m and £18.8 m, respectively.

A further sensitivity analysis for inpatient episodes costs with individual country results for England, Scotland and Northern Ireland produced similar results (Additional file 1: Appendix 7). However, due to the higher rates of inpatient episodes per head of population reported in Wales, sensitivity analysis which extrapolated all inpatient episode costs from the Welsh results raised the estimate to £147.0 m, i.e. approximately 70 % higher than the base case. Figures extrapolated from each of the other countries to Wales on the other hand ranged from £5.0 m to £5.5 m, or approximately 32–38 % lower (Additional file 1: Appendix 7).

## Discussion

We found that the prevalence of asthma varied widely depending on the definition used, ranging from 29.5 % (18.5 m people) for lifetime symptoms suggestive of asthma to 5.7 % (3.6 m) for those with active, clinician-diagnosed-and-treated asthma. Considerable care therefore needs to be taken in defining the populations being discussed and consistent use of the seven different definitions proposed in this paper should help greatly in this respect. We also found that, even with conservative assumptions, there was considerable morbidity, healthcare utilisation and costs such that asthma now costs the UK public sector well in excess of £1.1 bn per annum. The overwhelming majority of these costs are incurred in relation to prescribing in primary care for preventive treatments, but despite this, there were almost 100,000 inpatient episodes for asthma and over 1000 asthma deaths. These data suggest that particular focus is warranted on primary care to assess whether the most effective and cost-effective treatment strategies are consistently being employed [53], and on the development of innovative strategies for the prevention and early detection of asthma attacks.

We have produced the most comprehensive national work ever undertaken estimating the prevalence, care utilisation and financial costs of asthma in the UK. We scoped, obtained data from and interrogated 27 health and social care datasets from across the four nations of the UK, which either used well-defined sampling strategies (e.g. the national surveys) or covered large sections of the population (e.g. primary care databases) or indeed entire nations (e.g. hospital episode statistics and mortality data). We believe that our findings are therefore likely to be generalizable across the UK. Additional strengths come from the fact that we followed a pre-specified analysis plan and that we undertook a range of pre-specified sensitivity analyses to test our assumptions [7].

There are, however, a number of limitations that need to be considered. First, whilst we have undoubtedly made progress in addressing important data gaps previously identified (e.g. in relation to providing estimates for out-of-hours care, urgent care clinics, ambulance trips, A&E attendances and ICU admissions), some still exist, for example, in relation to out-patient clinic visits, presenteeism (i.e. attending work when unwell) [54], and absence from work to care for children [54]. Our results from A&E data marts may be underestimates because patients presenting with asthma exacerbations may not always have been coded with asthma (with terms such as shortness of breath or wheeze being used instead). We could not access reliable data on prescribing of Omalizumab, a biological agent used for severe, persistent asthma [55]. Given the above described limitations, our cost estimates should therefore be seen as minimum likely financial costs to the UK public sector. Second, whilst use of national surveys offers important insights into patient perspectives, these exclude the homeless, those living in institutional care and special populations (e.g. armed forces and prisoners). Third, many of the costs estimated in this study required extrapolation from one country to another (Additional file 1: Appendix 5). This process was undertaken by first rescaling based on differences in Office of National Statistics population estimates, then by annual prevalence of clinician-reported-diagnosed-and-treated (QOF) asthma between countries. While broader definitions of asthma would be expected to generate larger prevalence estimates within a country, we might expect their relative rates between countries to be similar and thus have minimally impacted this process. However, this assumption is impossible to test since no other definition of prevalence is uniformly measured within all four countries. It was not possible to account for differences in definitions of diagnosis of asthma (i.e. before uplift to national level estimates where necessary) as observed in differences between coding systems because basic resource use attributable to asthma is recorded using varying system coding based definitions (such as Read or ICD-10). The extrapolation process additionally accounted for differences in population by age and sex and prevalence in countries, but due to data limitations, could not account for other factors such as socioeconomic status/deprivation, ethnicity or disease severity profiles. It also makes the assumption that the rate of resource use per asthma patient is the same in all UK countries. We made efforts to cross-validate this assumption by comparing extrapolated results to known results, where possible. Although the majority of these exercises produced similar figures, extrapolating inpatient episode costs to and from Wales provided an exception due to the higher rate of inpatient episodes observed in Wales (Table 4). It is not possible to rule out similar issues where extrapolation could not be cross-validated.

The age-standardised prevalence and burden of asthma reported in our study are not easily compared to other estimates of asthma prevalence because (1) of differences in the age-groups, time periods and geographical settings studied [1, 3, 56–59]; (2) in contrast to many previous studies, we generated a number of estimates of asthma ‘prevalence’; and (3) DALYs have been reported in some previous studies (which was neither within the scope of this work nor was it possible given the data gaps identified). Our estimate of the proportion of medication cost broadly agrees with a systematic review which found that medications accounted for 38 % to 89 % of the total cost of asthma [5]. Although we have captured more data sources and costs, the increases to total costs were small and partially offset by more conservative prescribing assumptions. This is likely to explain why our costs are similar to a previous study in England and Wales that estimated spending at £754.4 m in 2000–02 [2], (£994.9 m at 2011–12 prices). They are also lower in Scotland than a previous study which estimated £98.1 m in 2003–05 (£117.0 m in 2011–12 prices) [3], again likely due to methodological differences and the more conservative approach used here. For example, only the burden of asthma as the main problem of the patient had been taken into account. Thus, for a patient with asthma and comorbidities which might have resulted in higher health and societal care utilisation, additional costs of care for comorbidities were not accounted for. It is therefore important that these estimates are not confused with burden of asthma, and it is clarified that these estimates are for burden of asthma in patients who utilised health and societal care when asthma was their main problem. Despite these differences, there is broad agreement that the UK has one of the highest asthma burdens in the world [1, 58, 60].

We have created a profile of asthma for the UK based on available data. This information will be useful to national and regional policymakers and health planners both in the UK and internationally since it can be used as a template for similar mapping of asthma in other nations. It should also be of considerable interest to respiratory physicians, GPs and the public both to consider the current level of health and social care utilisation and by serving as a benchmark for national and regional improvement efforts. Our work also has important implications for the academic community, particularly in relation to considering approaches to harmonising definitions and data collection procedures across the four nations of the UK [23], and in terms of finding novel ways of filling the outstanding data gaps. Finally, this work will also offer a number of potentially transferable lessons for generating robust national estimates of the epidemiology, care utilisation and costs of other long-term conditions.

## Conclusions

In summary, we have found that the UK continues to experience a very high disease and cost burden from asthma. Since much of the morbidity and mortality is considered potentially preventable, there is a need for ambitious national targets for reducing asthma exacerbations and the associated risks of hospitalisations and deaths.

## Additional file

Additional file 1:
**Appendix 1**. Read codes for asthma, version 2. **Appendix 2**. List of asthma medications by BNF codes. **Appendix 3**. Read codes for asthma, version 3. **Appendix 4**. Price weights and costings methodology. **Appendix 5**. Data sources and mapping used in economic modelling of the cost of asthma in theUK by member countries. **Appendix 6**. Cost modelling technical supplement. **Appendix 7**. Comparison of mapped inpatient episodes to known values for their respective countries. (DOCX 88 kb)
