# Supplementary material for: The epidemiology, healthcare and societal burden and costs of asthma in the UK and its member nations: analyses of standalone and linked national databases
Source: BMC Med. 2016 Aug 29;14(1):113. doi: 10.1186/s12916-016-0657-8 (PMC5002970; doi:10.1186/s12916-016-0657-8)
Supplement: Additional file 1: — Appendix 1. Read codes for asthma, version 2. Appendix 2. List of asthma medications by BNF codes. Appendix 3. Read codes for asthma, version 3. Appendix 4. Price weights and costings methodology. Appendix 5. Data sources and mapping used in economic modelling of the cost of asthma in theUK by member countries. Appendix 6. Cost modelling technical supplement. Appendix 7. Comparison of mapped inpatient episodes to known values for their respective countries. (DOCX 88 kb) [file 12916_2016_657_MOESM1_ESM.docx]

Additional File 1: Appendices 1-8

Appendix 1: Read codes for asthma, version 2

| **Code** | **Term** |
| --- | --- |
| H33.. | Asthma |
| H330. | Extrinsic (atopic) asthma |
| H3300 | Extrinsic asthma without status asthmaticus |
| H3301 | Extrinsic asthma with status asthmaticus |
| H330z | Extrinsic asthma NOS |
| H331. | Intrinsic asthma |
| H3310 | Intrinsic asthma without status asthmaticus |
| H3311 | Intrinsic asthma with status asthmaticus |
| H331z | Intrinsic asthma NOS |
| H332. | Mixed asthma |
| H333. | Acute exacerbation of asthma |
| H334. | Brittle asthma |
| H335. | Chronic asthma with fixed airflow obstruction |
| H33z. | Asthma unspecified |
| H33z0 | Status asthmaticus NOS |
| H33z1 | Asthma attack |
| H33z2 | Late-onset asthma |
| H33zz | Asthma NOS |
| 1780. | Aspirin induced asthma |
| 1781. | Asthma trigger - pollen |
| 1782. | Asthma trigger - tobacco smoke |
| 1783. | Asthma trigger - warm air |
| 1784. | Asthma trigger - emotion |
| 1785. | Asthma trigger - damp |
| 1786. | Asthma trigger - animals |
| 1787. | Asthma trigger - seasonal |
| 1788. | Asthma trigger - cold air |
| 1789. | Asthma trigger - respiratory infection |
| 178A. | Asthma trigger - airborne dust |
| 178B. | Asthma trigger - exercise |
| 1O2.. | Asthma confirmed |
| 66YK. | Asthma follow-up |
| 663.. | Asthma monitoring |
| 663N. | Asthma disturbing sleep |
| 663N0 | Asthma causing night waking |
| 663N1 | Asthma disturbs sleep weekly |
| 663N2 | Asthma disturbs sleep frequently |
| 663O. | Asthma not disturbing sleep |
| 663O0 | Asthma never disturbs sleep |
| 663P. | Asthma limiting activities |
| 663P0 | Asthma limits activities 1 to 2 times per month |
| 663P1 | Asthma limits activities 1 to 2 times per week |
| 663P2 | Asthma limits activities most days |
| 663Q. | Asthma not limiting activities |
| 663U. | Asthma management plan given |
| 663V. | Asthma severity |
| 663V0 | Occasional asthma |
| 663V1 | Mild asthma |
| 663V2 | Moderate asthma |
| 663V3 | Severe asthma |
| 663W. | Asthma prophylactic medication used |
| 663d. | Emergency asthma admission since last appointment |
| 663e. | Asthma restricts exercise |
| 663f. | Asthma never restricts exercise |
| 663h. | Asthma - currently dormant |
| 663j. | Asthma - currently active |
| 663m. | Asthma accident and emergency attendance since last visit |
| 663n. | Asthma treatment compliance satisfactory |
| 663p. | Asthma treatment compliance unsatisfactory |
| 663q. | Asthma daytime symptoms |
| 663r. | Asthma causes night symptoms 1 to 2 times per month |
| 663s. | Asthma never causes daytime symptoms |
| 663t. | Asthma causes daytime symptoms 1 to 2 times per month |
| 663u. | Asthma causes daytime symptoms 1 to 2 times per week |
| 663v. | Asthma causes daytime symptoms most days |
| 663w. | Asthma limits walking up hills or stairs |
| 663x. | Asthma limits walking on the flat |
| 663y. | Number of asthma exacerbations in past year |
| 66YJ. | Asthma annual review |
| 66YE. | Asthma monitoring due |
| 66Y5. | Change in asthma management plan |
| 66Y9. | Step up change in asthma management plan |
| 66YA. | Step down change in asthma management plan |
| 66Yq. | Asthma causes night time symptoms 1 to 2 times per week |
| 66Yr. | Asthma causes symptoms most nights |
| 66Yp. | Asthma review using Roy College of Physicians three questions |
| 8CMA0 | Patient has a written asthma personal action plan |
| 8B3j. | Asthma medication review |
| 8793. | Asthma control step 0 |
| 8794. | Asthma control step 1 |
| 8795. | Asthma control step 2 |
| 8796. | Asthma control step 3 |
| 8797. | Asthma control step 4 |
| 8798. | Asthma control step 5 |
| 9OJ1. | Attends asthma monitoring |
| 9OJA. | Asthma monitoring check done |
| 173d. | Work aggravated asthma |
| 663e0 | Asthma sometimes restricts exercise |
| 663e1 | Asthma severely restricts exercise |
| 66YC. | Absent from work or school due to asthma |
| 66YK. | Asthma follow-up |
| 66YP. | Asthma night-time symptoms |
| 66YQ. | Asthma monitoring by nurse |
| 66YR. | Asthma monitoring by doctor |
| 66Ys. | Asthma never causes night symptoms |
| 66Yu. | Number days absent from school due to asthma in past 6 month |
| 8791. | Further asthma - drug prevent. |
| 8CR0. | Asthma clinical management plan |

Appendix 2: List of asthma medications by BNF codes

*Source: BNF March to September 2013; list of immunosuppresants from AUK-PNG working group, the BNF codes of immunosuppresants and the ‘other conditions’ they are used for are derived from Monthly Index of Medical Specialities (MIMS). Age criteria checked with BNF children.*

| **BNF section name** | **BNF sub-section name** | **BNF sub-sub-section name** | **BNF sub-sub-sub section number** | **BNF sub-sub-sub-section name** | **BNF generic drug name** | **Drug brand name** | **Form** | ***EXCLUDE*** | **Asthma** | **COPD** | **Other conditions [reversible airways obstruction (RAO)]** | **Age criteria** |
| --- | --- | --- | --- | --- | --- | --- | --- | --- | --- | --- | --- | --- |
| Bronchodilators | Adrenoceptor agonists | Selective beta_2_ agonists | 03010101 | Short acting beta_2_ agonists | Salbutamol -Albuterol | Salbutamol - Non-proprietary, AirSalb, Salamol, Salamol Steri-Neb, Salapin, | tablet,  injection (subcutaneous, intramuscular, intravenous),  infusion (intravenous,  inhalation of aerosol/powder/nebulised solution (inhalation) |  | ✓ | ✓ | RAO, premature labour | all age |
|  |  |  |  |  |  | Ventmax SR | capsule |  | ✓ | ✓ |  | all age |
|  |  |  |  |  |  | Ventolin | syrup, injection, accuhaler (dry powder for inhalation), respirator solution (for use with nebuliser or ventilator) |  | ✓ | ✓ |  | all age |
|  |  |  |  |  |  | Airomir | aerosol inhalation | | ✓ | ✓ |  | all age |
|  |  |  |  |  |  | Asmasal Clickhaler | dry powder for inhalation | | ✓ | ✓ |  | all age |
|  |  |  |  |  |  | Easyhaler Salbutamol | dry powder for inhalation | | ✓ | ✓ |  | all age |
|  |  |  |  |  |  | Pulivinal Salbutamol | dry powder for inhalation | | ✓ | ✓ |  | all age |
|  |  |  |  |  |  | Salamol Esi-Breathe | aerosol inhalation | | ✓ | ✓ |  | all age |
|  |  |  |  |  |  | Salbulin Novolizer | dry powder for inhalation | | ✓ | ✓ |  | all age |
|  |  |  |  |  | Bambuterol hydrochloride | Bambec | tablet |  | ✓ | ✓ | RAO |  |
|  |  |  |  | Long acting beta_2_ agonists (LABA) | Formoterol (eformoterol) - Formoterol/eformoterol fumarate | Formoterol -Non-proprietary | dry powder for inhalation | | ✓ | ✓ | RAO in patients requiring long term regular bronchodilator therapy | 6 onwards |
|  |  |  |  |  |  | Atimos modulite | aerosol inhalation | | ✓ | ✓ |  | 12 onwards |
|  |  |  |  |  |  | Foradil | dry powder for inhalation | | ✓ | ✓ |  | 6 onwards |
|  |  |  |  |  |  | Oxis Turbohaler | dry powder inhaler | | ✓ | ✓ |  | 6 onwards |
|  |  |  |  |  |  |  |  | Indaceterol -Onbrez Breezhaler | ✓ | ✓ |  |  |
|  |  |  |  |  | Salmeterol | Salmeterol - Non-proprietary, Neovent, Xinofoate, | aerosol inhalation | | ✓ | ✓ | RAO in patients requiring long term regular bronchodilator therapy | children |
|  |  |  |  |  |  | Serevent - Accuhaler, Diskhaler, Evohaler | dry powder for inhalation (Accuhaler, Diskhaler), aerosol inhalation (Evohaler) |  | ✓ | ✓ |  | children |
|  |  |  |  |  | Terbutaline Sulfate | Bricanyl | tablet, syrup, injection (subcutaneous/slow intravenous, solution (continous intravenous infusion) |  | ✓ | ✓ | RAO, premature labour | all age |
|  |  |  |  |  |  | Bricanyl Turbohaler | powder for inhalation (Turbohaler) |  | ✓ | ✓ |  | all age |
|  |  |  |  |  |  | Bricanyl Respules | nebuliser solution |  | ✓ | ✓ |  | all age |
|  |  |  |  |  |  |  |  | Other adrenoceptor agonists 03010102- Adrenaline (epinephrine) - Adrenaline 1 in 1000, Epipen, Epipen Junior, Jext |  | ✓ | emergency treatment of acute allergic and anaphylactic rections, angioedema, cardiopulmonary resuscitation, severe croup |  |
|  |  |  |  |  |  |  |  | Other adrenoceptor agonists 03010102- Ephedrine hydrochloride -Ephedrine hydrochloride - Non-proprietary |  | ✓ | RAO |  |
| Bronchodilators | Antimuscarinic bronchodilators |  |  |  |  |  |  | Aclidinium bromide -Eklira Genuair |  | ✓ |  |  |
|  |  |  |  |  |  |  |  | Glycopyrronium -Seebri Breezhaler |  | ✓ |  |  |
|  |  |  |  |  | Ipratropium bromide | Ipratropium bromide - Non-proprietary | nebuliser solution |  | ✓ | ✓ | RAO, rhinitis | all age |
|  |  |  |  |  |  | Atrovent | aerosol inhalation |  | ✓ | ✓ |  | all age |
|  |  |  |  |  |  | Ipratropium Steri-Neb | nebuliser solution |  | ✓ | ✓ |  | all age |
|  |  |  |  |  |  | Respontin | nebuliser solution |  | ✓ | ✓ |  | all age |
|  |  |  |  |  |  |  |  | Tiotropium - Spiriva |  | ✓ |  |  |
|  | Theophylline |  |  |  | Theophylline | Neulin SA | tablets |  | ✓ | ✓ | RAO | all age |
|  |  |  |  |  |  | Slo-Phyllin | capsules |  | ✓ | ✓ |  | 2 onwards |
|  |  |  |  |  |  | Uniphyllin Continus | tablets |  | ✓ | ✓ |  | all age |
|  |  |  |  |  | Aminophylline | Aminophylline - Non-proprietary | injection |  | ✓ | ✓ | RAO, bronchospasm associated with chronic bronchitis | all age |
|  |  |  |  |  |  | Phyllocontin Continus | tablets |  | ✓ | ✓ |  |  |
|  | Compound bronchodilator preparations |  |  |  |  | Ipratropium bromide with Salbutamol - Non-proprietary, Salipraneb, Ipramol | nebuliser solution |  | ✓ | ✓ |  | over 12 |
|  |  |  |  |  |  | Combivent | nebuliser solution |  | ✓ | ✓ |  | over 12 |
|  | Peak flow meters, inhaler devices and nebulisers |  |  | Peak flow meters | Standard range peak flow meter | AirZone, Medi, MicroPeak, Mini-Wright, Personal Best, Piko-1, Pinnacle, Pocketpeak, Vitalograph |  |  | ✓ |  |  | over 5 |
|  |  |  |  |  | Low range peak flow meter | Medi, Mini-Wright, Pocketpeak |  |  | ✓ |  |  | over 5 |
|  |  |  |  | Inhaler devices | pressurised metered dose inhalers, breath-actuated inhalers and dry powder inhalers |  |  |  | ✓ | ✓ |  | in children, age not stated |
|  |  |  |  | Spacer devices | A2A Spacer, Able Spacer, AeroChamber Plus, Babyhaler, Haleraid, Optichamber, Vortex Spacer, Pocket Chamber, Volumatic |  |  |  | ✓ | ✓ |  | in children, age not stated |
|  |  |  |  | Nebulisers and compressors | Nebuliser diluent | Sodium chloride - Non-proprietary, Saline Steripoule, Saline Steri-Neb |  |  | ✓ | ✓ | RAO, cystic fibrosis, severe croup, pneumocystis pneumonia | in children, age not stated |
| Corticosteroids | Beclometasone dipropionate |  |  |  |  | Beclometasone - Non-proprietary, Pulvinal, Beclometasone dipropionate, Easyhaler Beclometasone dipropionate | dry powder inhalation | | ✓ | ✓ |  | in children, age not stated |
|  |  |  |  |  |  | Asmabec Clickhaler | dry powder inhalation | | ✓ | ✓ |  | 6 onwards |
|  |  |  |  |  |  | Becodisks | dry powder inhalation | | ✓ | ✓ |  | 5 onwards |
|  |  |  |  |  |  | Clenil modulite | aerosol inhalation | | ✓ | ✓ |  | 2 onwards |
|  |  |  |  |  |  | Qvar | aerosol inhalation | | ✓ | ✓ |  | over 12 |
|  |  |  |  | Compound preparations |  | Fostair | aerosol inhalation | | ✓ | ✓ |  | over 18 |
|  | Budesonide |  |  | Budesonide |  | Budesonide - Non-proprietary, Easyhaler Budesonide | dry powder inhalation | | ✓ | ✓ |  | 6 onwards |
|  |  |  |  |  |  | Budelin Novolizer | dry powder inhalation | | ✓ | ✓ |  | 6 onwards |
|  |  |  |  |  |  | Pulmicort Turbohaler | dry powder inhalation | | ✓ | ✓ |  | 5 onwards |
|  |  |  |  | Compound preparations |  | Symbicort,  Symbicort 100/6 Turbohaler, Symbicort 200/6 Turbohaler, Symbicort 400/12 Turbohaler | dry powder inhalation | | ✓ | ✓ |  | 6 onwards |
|  | Ciclesonide |  |  |  |  | Alvesco | aerosol inhalation | | ✓ | ✓ |  | 12 onwards |
|  | Fluticasone propionate |  |  |  |  | Flixotide Accuhaler,  Flixotide Evohaler, Nebules | dry powder for inhalation (Accuhaler), aerosol inhalation (Evohaler) |  | ✓ | ✓ |  | 5 onwards |
|  |  |  |  | Compound preparations |  | Flutiform | aerosol inhalation | | ✓ | ✓ |  | 12 onwards |
|  |  |  |  |  |  | Seretide,  Seretide 100 Accuhaler, Seretide 250 Accuhaler,  Seretide 500 Accuhaler,  Seretide 125 Evohaler,  Seretide 250 Evohaler | dry powder for inhalation (Accuhaler), aerosol inhalation (Evohaler) |  | ✓ | ✓ |  | 5 onwards |
|  | Mometasone Furoate |  |  |  |  | Asmanex Twisthaler | dry powder inhalation | | ✓ | ✓ |  | 12 onwards |
| Cromoglicate and related therapy, leukotriene receptor antagonist | Cromoglicate and related therapy |  |  |  | Sodium cromoglicate/ cromoglycate | Intal CFC-free Inhaler | aerosol inhalation |  | ✓ | ✓ | food allergy, allergic conjunctivitis, allergic rhinitis | 5 onwards |
|  |  |  |  |  | Nedocromil sodium | Tilade CFC-free Inhaler | aerosol inhalation | | ✓ | ✓ |  | 5 onwards |
|  | Leukotriene receptor antagonist |  |  |  | Montelukast | Singulair | tablets |  | ✓ |  | seasonal allergic rhinitis | All age |
|  |  |  |  |  | Zafirlukast | Accolate | tablets |  | ✓ | ✓ |  | 12-18 yrs |
|  |  |  |  |  |  |  |  | Phosphodiesterase type-4 inhibitors -Roflumilast, Daxas |  |  |  |  |
|  |  |  |  |  |  |  |  | Antihistamines 030401,  allergen immunotherapy 030402 *except* Omalizumab,  allergic emergencies 030403 |  |  |  |  |
|  |  | Omalizumab ONLY | |  |  | Xolair | injection |  | ✓ |  |  | over 6 years |
| Immunosuppresants |  |  |  |  | Prednisolone | Predsol, Minims Prednisolone, Pred Forte, Deltacortil, Deltastab, Prednisolone, Prednisolone Soluble Tablets, Prednisolone Gastroresistant Tablets, Predsol Enema, | drops, tablets, injections |  | ✓ |  | ear conditions, eye inflammation, inflammatory and allergic disorders, croup, ulcerative colitis, Crohn's disease |  |
|  |  |  |  |  | Methotrexate | Maxtrex, Methotrexate, Metoject | tablet, injection |  | ✓ |  | antineoplastics, rheumatoid arthritis, other auto-immune disorders, psoriasis, seborrhoea etc | all age |
|  |  |  |  |  | Ciclosporin | Capimune, Capsorin, Deximune, Neoral, Neoral capsules, Sandimmun | oral solutions, capsules |  | ✓ |  | rheumatoid arthritis, other auto-immune disorders, organ transplantation, psoriasis, seborrhoea etc, renal disorders, Inflammatory bowel disease | all age |
|  |  |  |  |  | Azathioprine | Azathioprine, Imuran, Imuran injection | tablet, powder in vial |  | ✓ |  | rheumatoid arthritis, other auto-immune disorders, organ transplantation | all age |

Appendix 3: Read codes for asthma, version 3

Includes version 2 codes above and the following

| **Code** | **Term** |
| --- | --- |
| XE0YX | Asthma NOS |
| Xa9zf | Acute asthma |
| XE0YW | Asthma attack |
| X1020 | Pollen asthma |
| X1026 | Baker's asthma |
| X102Y | Cardiac asthma |
| Ua1AX | Brittle asthma |
| XaIOV | Asthma finding |
| XaINi | Number of times bronchodilator used in one week |
| XaINj | Number of times bronchodilator used in 24 hours |
| XaIww | Asthma trigger |
| XaLIm | Asthma trigger - respiratory infection |
| XaLIn | Asthma trigger - seasonal |
| XaLIr | Asthma trigger - animals |
| XaLJS | Asthma trigger - cold air |
| XaLJT | Asthma trigger - damp |
| XaLJU | Asthma trigger - emotion |
| XaObi | Asthma trigger - airborne dust |
| XaObj | Asthma trigger - exercise |
| XaObk | Asthma trigger - pollen |
| XaObl | Asthma trigger - tobacco smoke |
| XaObm | Asthma trigger - warm air |
| XaYja | Asthma trigger - wind |
| XaYpF | Asthma trigger - perfume |
| X101x | Allergic asthma |
| XE2Nb | Asthma monitored |
| XaLPE | Nocturnal asthma |
| X101t | Childhood asthma |
| XaIer | Asthma follow-up |
| XaInC | Suspected asthma |
| XaIuG | Asthma confirmed |
| X1027 | Colophony asthma |
| XM1Xb | Asthma monitoring |
| X101u | Late onset asthma |
| XM0s2 | Asthma attack NOS |
| X1025 | Industrial asthma |
| X102D | Status asthmaticus |
| XE0YV | Status asthmaticus NOS |
| XE0YT | Non-allergic asthma |
| X1025 | Occupational asthma |
| X102D | Acute severe asthma |
| X101z | Allergic asthma NEC |
| X1023 | Drug-induced asthma |
| XaQHq | Asthma control test |
| Xa0lZ | Asthmatic bronchitis |
| Xa8Hn | Asthma control steps |
| XaIeq | Asthma annual review |
| XaLJT | Asthma trigger - damp |
| XaYja | Asthma trigger - wind |
| XaIRN | Asthma monitoring due |
| X1020 | Hay fever with asthma |
| X1028 | Grain worker's asthma |
| XE0YQ | Allergic atopic asthma |
| XaJFG | Aspirin-induced asthma |
| Xa1hD | Exacerbation of asthma |
| XE0YV | Status asthmaticus NOS |
| XaBU2 | Asthma monitoring call |
| XaKdk | Work aggravated asthma |
| X1029 | Sulphite-induced asthma |
| XaIIZ | Asthma daytime symptoms |
| XaObk | Asthma trigger - pollen |
| XaLJU | Asthma trigger - emotion |
| XaYpF | Asthma trigger - perfume |
| XaBU3 | Asthma monitoring status |
| XE2Na | Asthma monitoring admin. |
| XaBAQ | Recent asthma management |
| XaIfK | Asthma medication review |
| XaDvK | Asthma - currently active |
| XaJtu | Referral to asthma clinic |
| X1021 | Allergic non-atopic asthma |
| XaIoE | Asthma night-time symptoms |
| XaIu5 | Asthma monitoring by nurse |
| XaDvL | Asthma - currently dormant |
| XaIu6 | Asthma monitoring by doctor |
| XaQig | Asthma control questionnaire |
| XaJ2A | Did not attend asthma clinic |
| XE2Nb | Asthma monitoring check done |
| XaR8K | Did not attend asthma review |
| XM1U3 | Asthma clinic administration |
| XaY2q | Seen in school asthma clinic |
| Xa1hD | Acute exacerbation of asthma |
| XaIQ4 | Change in asthma management plan |
| X102G | Asthmatic pulmonary eosinophilia |
| XaINg | Asthma limits walking on the flat |
| XaXZp | Asthma causes symptoms most nights |
| XaY2V | Asthma never causes night symptoms |
| XaXZx | Asthma limits activities most days |
| XaYb8 | Asthma self-management plan agreed |
| XaYZB | Asthma self-management plan review |
| XaQig | ACQ - Asthma control questionnaire |
| XaJ2A | DNA - Did not attend asthma clinic |
| X1022 | Intrinsic asthma with asthma attack |
| X101y | Extrinsic asthma with asthma attack |
| XaINa | Asthma never causes daytime symptoms |
| X1024 | ASA - Aspirin-sensitive asthma triad |
| XaJuw | Does not have asthma management plan |
| XaQij | Under care of asthma specialist nurse |
| XaLIm | Asthma trigger - respiratory infection |
| Xaa7Q | No asthma trigger identified by subject |
| XaIIX | Asthma treatment compliance satisfactory |
| XE0YS | Extrinsic asthma with status asthmaticus |
| XaIQD | Step up change in asthma management plan |
| XaINd | Asthma causes daytime symptoms most days |
| XE0YU | Intrinsic asthma with status asthmaticus |
| XaIR3 | Absent from work or school due to asthma |
| XaINf | Asthma limits walking up hills or stairs |
| XaRFj | Health education - asthma self-management |
| XaQih | Mini asthma quality of life questionnaire |
| XaIQE | Step down change in asthma management plan |
| XaIIY | Asthma treatment compliance unsatisfactory |
| X1024 | Aspirin-sensitive asthma with nasal polyps |
| XE0YR | Extrinsic asthma without status asthmaticus |
| XaINh | Number of asthma exacerbations in past year |
| Xaa7B | Chronic asthma with fixed airflow obstruction |
| Xa544 | Accidental poisoning by herbal asthma mixture |
| XaNKw | Royal College of Physicians asthma assessment |
| XaJ4W | Exception reporting: asthma quality indicators |
| XaXZu | Asthma limits activities 1 to 2 times per week |
| XaRFk | Health education - structured asthma discussion |
| XaXZs | Asthma limits activities 1 to 2 times per month |
| XaRFi | Patient has a written asthma personal action plan |
| XaINZ | Asthma causes night symptoms 1 to 2 times per month |
| XaINc | Asthma causes daytime symptoms 1 to 2 times per week |
| XaINb | Asthma causes daytime symptoms 1 to 2 times per month |
| XaXZm | Asthma causes night time symptoms 1 to 2 times per week |
| XaIIW | Asthma accident and emergency attendance since last visit |
| Xa546 | Poisoning by herbal asthma mixture of undetermined intent |
| XaJ50 | Excepted from asthma quality indicators: Informed dissent |
| XaXa0 | Royal College of Physicians asthma assessment three questions score |
| XaJ4z | Excepted from asthma quality indicators: Patient unsuitable |
| XaX3n | Asthma review using Royal College of Physicians three questions |
| XaYZh | Number of days absent from school due to asthma in past 6 months |
| XaRFl | Health education - structured patient focused asthma discussion |

**Appendix 4: Price weights and costings methodology**

| **Variable** | **Value** | | | | | **Notes/source** |
| --- | --- | --- | --- | --- | --- | --- |
|  |  |  | |  | |  |
| GP consultations | £40 | per | | consultation | | Personal Social Services Research Unit (PSSRU)[[1](#_ENREF_1)] |
| Practice nurse consultation | £14 | per | | consultation | | Personal Social Services Research Unit (PSSRU)[[1](#_ENREF_1)] |
| Out-of-hour calls | £28·38 | per | | call | | £25·53 in 2007/8,[[2](#_ENREF_2)] inflated to 2011/12 using HSCS index[[1](#_ENREF_1)] |
| Community prescribing: | Micro-costed | per | | Prescription | |  |
| *Scotland* | Costing at prescription level (Gross Ingredient Costs including discount for broken bulk) based on pre-calculated internal price weights· | | | | | Prescribing Information Systems data internal price estimates· |
| *Wales* | Costing at prescription level based on Net Ingredient Costs from British National Formulary· | | | | | British National Formulary 2012[[3](#_ENREF_3)] |
| Ambulance trips | £294 | per | | journey | | £257 in 2006/7,[[4](#_ENREF_4)] inflated to 2011/12 using HSCS index[[1](#_ENREF_1)] |
| A&E visit | £108 | per | | visit | | Department of Health (DoH), NHS reference costs[[4](#_ENREF_4)] |
| Inpatient episodes: | Micro-costed | per | | Episode | |  |
| *England, Northern Ireland and Scotland* | Costing at episode level based on admission type (day case, elective, non-elective short, non-elective long)and HRG4 code | | | | | Department of Health (DoH), NHS reference costs[[4](#_ENREF_4)] |
| *Wales* | Costing per episode based on admission type (day case, elective, non-elective short, non-elective long)· | | | | | Source: English per episode cost estimates derived as above· |
| ICU episodes: Children | Average cost per day (Males: £2,299·23, Females: £2,304·86) multiplied by length of stay based on HRG4 code based costing of subsample of episodes | | | | | Department of Health (DoH), NHS reference costs[[4](#_ENREF_4)] |
| ICU episodes: Adults | £2,324·46 per episode | | | | | Weighted average cost of a critical care stay for· 0 or 1 organ supported[[4](#_ENREF_4)] |
| Disability Living Allowance: |  | | | | |  |
| *England, Scotland and Wales* | Aggregated level costs provided only, these have been derived from absolute values of public records of benefit payment records | | | | | Department of work and pensions |
| *Northern Ireland* | Aggregated level costs provided only, these have been derived from absolute values of public records of benefit payment record | | | | | Department for Social Development |
|  |  | |  | |  |  |

**Appendix 5: Data sources and mapping used in economic modelling of the cost of asthma in the UK by member countries**

| **Variable** | **Countries** | | | |
| --- | --- | --- | --- | --- |
|  | **England** | **Northern Ireland** | **Scotland** | **Wales** |
| **Annual prevalence of clinician-reported-diagnosed-and-treated asthma absolute values** | Quality of Outcomes Framework | Quality of Outcomes Framework | Quality of Outcomes Framework | Quality of Outcomes Framework |
| **Population distributions by age and sex groupings in people aged 40 years and less** | Health Survey for England 2010 | Proportions assumed to be equal to those from Health Survey for England 2010 | Scottish Health Survey 2010 | SAIL GP database  2010/11 |
| **Population distributions by age and sex groupings in people above 40 years** | Health Survey for England 2010 | Health Survey for Northern Ireland 2010 | Scottish Health Survey 2010 | SAIL GP database 2010/11 |
|  |  |  |  |  |
| **GP consultations** | Mapped from Scotland | Mapped from Scotland | Practice Team Information | Mapped from Scotland |
| **Practice Nurse consultations** | Mapped from Scotland | Mapped from Scotland | Practice Team Information | Mapped from Scotland |
| **Community prescribing in people aged 40 years and less** | Mapped from Wales | Mapped from Wales | Prescribing Information System | SAIL GP database 2010/11 |
| **Community prescribing in people aged over 40 years** | Mapped from Wales | Mapped from Wales | Mapped from Wales | SAIL GP database 2010/11 |
| **Calls to out-of-hours** | Mapped from Scotland | (Not applicable) | NHS 24 | Mapped from Scotland |
| **Ambulance trips** | Mapped from Scotland | Mapped from Scotland | Scottish Ambulance Service | Mapped from Scotland |
| **A&E** | Mapped from Scotland | Extract of data from Belfast HSCT | A&E Datamart | Mapped from Scotland |
| **Hospital episodes** | Hospital Episode Statistics | Department of Health, Social Services and Public Safety Northern Ireland | Scottish Morbidity Records for inpatient and day-cases | Patient Episode Data for Wales |
| **ICU episodes in children** | Paediatric Intensive Care Audit Network | Paediatric Intensive Care Audit Network | Paediatric Intensive Care Audit Network | Paediatric Intensive Care Audit Network |
| **ICU episodes in adults** | Intensive Care National Audit and Research Centre | Intensive Care National Audit and Research Centre | Scottish Intensive Care Society Audit Group | Intensive Care National Audit and Research Centre |
| **Disability living allowance** | Department of Work and Pensions | Department for Social Development in Northern Ireland | Department of Work and Pensions | Department of Work and Pensions |

**Appendix 6: Cost modelling technical supplement**

**Health and societal care costs of asthma**

Healthcare costs were estimated from an NHS perspective based on healthcare utilisation. Appendix 5 summarises the costing methodology applied to each resource type and the associated variables. Where possible cost estimates of the resource being measured were taken from any cost estimates inherent in its dataset. However for the majority of cases, standard UK price weights were applied. All costs were estimated from a base year of 2011/12 applying appropriate inflation indices where required (Appendix 4).

**Mapping of resource use from one country to another**

Where data on a particular form of resource use was available in one member country but not in another, the cost associated with the resource in the country for which data was unavailable was estimated by mapping the total costs associated with the resource in the country for which data was available. Table 1 shows the data sources used for each resource type in each country.

This mapping was undertaken adjusting for the age and sex distributions of the population as a whole, based on the population estimates, and the prevalence of asthma in that country (also adjusting for age and sex distributions). The only comparable prevalence figures available in all four of the UK member countries to make this adjustment are those from QOF. However the QOF figures are unable to provide prevalence estimates broken down by age and sex due to the way the data are collected. In order to allow the mapping process to take into account any differences in the distributions of asthma sufferers in each country, the total number of asthma sufferers in each country were broken down into age and sex groupings based on the proportion of asthma suffers reported in the health surveys for each country, except Wales where these were based on the SAIL GP data for current treated asthma due to the considerably larger sample size that was available.

When extrapolating a particular form of cost estimate of from one country to another, the cost estimates for a given age-sex grouping per head of population were first extrapolated to population level estimates within their host country, if they did not already represent a complete sample. These were then divided by the respective age-sex group of the adjusted annual prevalence of clinician-reported-diagnosed-and-treated (QOF) estimates of asthma sufferers in the home country and multiplied by the equivalent in the country being mapped to. This approach effectively rescales on each country’s absolute population size, relative (as opposed to absolute) annual prevalence of clinician-reported-diagnosed-and-treated (QOF) prevalence and the age and sex distributions of each within each country. However it is unable to account for differences between countries in terms of a number of other likely important factors, in particular the profile of the severity of disease, socioeconomic profiles of the population, environmental factors, care pathways or availability or quality of healthcare facilities.

Unfortunately the data available did not facilitate adjustment for these factors and they remain a limitation of both the study and the available data stock on asthma, though some of these elements could potentially be overcome if access to some of the larger GP databases (which were unfortunately beyond the budget of this project) were to be utilised. Further work is being undertaken to investigate methodological approaches which might overcome some of these issues in the future as part of the UK Asthma Observatory.

**Economic modelling**

An economic model of the cost of asthma in the UK and its member countries was built in Microsoft Excel 2010. This was used to:

1. Apply the mapping techniques and synthesis of data with wider literature and price weights,
2. Sum up the cost estimates into the required groupings (e.g. NHS costs, wider societal costs each by age and gender groupings described above), and
3. estimate 95% CI around the joint distributions of each total cost estimate.

Confidence Interval Estimation

95% CIs around cost totals were estimated by bootstrap using the percentile method with a 5% alpha [[5](#_ENREF_5)]. This process involves applying probability distributions to each parameter in the model based on method of moment estimators [[6](#_ENREF_6)]. Following recommendations in standard modelling guidance [[6](#_ENREF_6)], the uncertainty around prevalence estimates were simulated using beta distributions and uncertainty around cost estimates were simulated using gamma distributions or normal distributions where sample sizes were large and central limit theorem was expected to hold. Model parameters were then varied simultaneously by random draws from these parameters and ran through the model to capture the joint distribution over all model parameters generating a sample cost. The resulting cost estimate from each sample (called a replicate) were then saved and a new replicate generated. Random samples in each replicate are drawn “with replacement” which is to say it is possible for the same random draw to occur for a given parameter or parameters in different replicates. The results for all replaces are then ranked and the 100*([alpha]/2) and 100*(1-([alpha]/2)) values were taken as the lower and upper confidence interval respectively [[5](#_ENREF_5)]. 10,000 replicates were used in this process.

It is important to be aware when using these confidence intervals that such an approach does not account for methodological uncertainty for example the assumption that rates of utilisation in the country being mapped from are equal to the rates of utilisation of the same resource in the country being mapped too. These confidence intervals only capture the uncertainty around the individual parameters going into the bootstrapping process, such as the interval around the prevalence estimate and the rate of utilisation. Hence the confidence intervals provided are subject to the same assumptions of the data they are derived from. More sophisticated approaches which may allow formal integration of such uncertainty are being investigated for future work in this area as part of the UK Asthma Observatory.

**Appendix 7: Comparison of mapped inpatient episodes to known values for their respective countries**

| **Result Country** | **Base Case** | **Country mapped from** | | | |
| --- | --- | --- | --- | --- | --- |
|  | **(£000’s)** | **England**  **(£000’s)** | **Northern Ireland**  **(£000’s)** | **Scotland**  **(£000’s)** | **Wales**  **(£000’s)** |
| **England** | 69,162 | 69,162 | 65,636 | 70,730 | 122,373 |
| **Northern Ireland** | 2,064 | 2,312 | 2,064 | 2,278 | 4,158 |
| **Scotland** | 6,342 | 6,708 | 6,178 | 6,342 | 12,381 |
| **Wales** | 8,128 | 5,255 | 5,018 | 5,501 | 8,128 |
| **UK** | **85,696** | **83,437** | **78,895** | **84,851** | **147,040** |

Bold text indicates base case result as used in the model

**Appendix 8: Data publicly available online**

1. Quality and Outcomes Framework (QOF), obtainable for

- England from [http://www.hscic.gov.uk/QOF](http://www.hscic.gov.uk/qof),
- Scotland from <http://www.isdscotland.org/Health-Topics/General-Practice/Quality-And-Outcomes-Framework/>,
- Wales from <http://gov.wales/statistics-and-research/general-medical-services-contract/?tab=previous&lang=en>,
- Northern Ireland from [https://www.health-ni.gov.uk/publications/quality-and-outcomes-framework-QOF-achievement-data-201112](https://www.health-ni.gov.uk/publications/quality-and-outcomes-framework-qof-achievement-data-201112)

1. national health surveys, obtainable for

- England from <https://discover.ukdataservice.ac.uk/catalogue/?sn=6986>,
- Scotland from <https://discover.ukdataservice.ac.uk/catalogue/?sn=6987>,
- Wales from <http://gov.wales/statistics-and-research/welsh-health-survey/?tab=previous&lang=en>,
- Northern Ireland from <https://www.health-ni.gov.uk/articles/health-survey-northern-ireland>

**References**

1. Curtis L, Personal Social Services Research Unit (PSSRU) University of Kent. Unit Costs of Health and Social Care 2012. 2012 http://www.pssru.ac.uk/project-pages/unit-costs/2012/ Accessed: 7 March 2016.

2. UK Parliament Commons Debates -Daily Hansard - Written Answers. NHS Direct: Operating Costs. 21 Oct 2008 : Column 314W 2008 http://www.publications.parliament.uk/pa/cm200708/cmhansrd/cm081021/text/81021w0033.htm#column_314W Accessed: 8 March 2016.

3. British National Formulary (BNF). 2012 www.bnf.org Accessed: 3 March 2016.

4. Department of Health (DoH). NHS reference costs 2011-12 https://www.gov.uk/government/publications/nhs-reference-costs-financial-year-2011-to-2012 Accessed: 3 March 2016.

5. Briggs AH, Wonderling DE, Mooney CZ: Pulling cost-effectiveness analysis up by its bootstraps: a non-parametric approach to confidence interval estimation. *Health Econ* 1997, 6:327-340.

6. Briggs A, Claxton K, Sculpher M: Decision modelling for health economic evaluation: Oxford University Press; 2006.
